# Time Series Analysis of Hemorrhagic Fever with Renal Syndrome: A Case Study in Jiaonan County, China

**DOI:** 10.1371/journal.pone.0163771

**Published:** 2016-10-05

**Authors:** Shujuan Li, Wei Cao, Hongyan Ren, Liang Lu, Dafang Zhuang, Qiyong Liu

**Affiliations:** 1 State Key Laboratory of Resources and Environmental Information System, Institute of Geographical Sciences and Natural Resources Research, Chinese Academy of Sciences, 11A Datun Road, Chaoyang District, Beijing, 100101, China; 2 College of Resources and Environment, University of Chinese Academy of Sciences, No. 19 Yuquan Road, Beijing, 100049, China; 3 State Key Laboratory for Infectious Diseases Prevention and Control, National Institute for Communicable Disease Control and Prevention, China CDC, 5 Changbai Road, Changping, Beijing, 102206, China; University of Minnesota, UNITED STATES

## Abstract

Exact prediction of Hemorrhagic fever with renal syndrome (HFRS) epidemics must improve to establish effective preventive measures in China. A Seasonal Autoregressive Integrated Moving Average (SARIMA) model was applied to establish a highly predictive model of HFRS. Meteorological factors were considered external variables through a cross correlation analysis. Then, these factors were included in the SARIMA model to determine if they could improve the predictive ability of HFRS epidemics in the region. The optimal univariate SARIMA model was identified as (0,0,2)(1,1,1)_12_. The R^2^ of the prediction of HFRS cases from January 2014 to December 2014 was 0.857, and the Root mean square error (RMSE) was 2.708. However, the inclusion of meteorological variables as external regressors did not significantly improve the SARIMA model. This result is likely because seasonal variations in meteorological variables were included in the seasonal characteristics of the HFRS itself.

## Introduction

Hemorrhagic fever with renal syndrome (HFRS) is a zoonosis caused by different species of Hantavirus, Hantaan virus (HNTV) transmitted by the striped field mouse (Apodemus agrarius), Seoul virus (SEOV) transmitted by the Norway rat (Rattus norvegicus), resulting in high fever and varying degrees of renal damage and hemorrhaging[1]. Approximately 90% of the world’s cases have been reported in China [2], with 10,000 cases annually in mainland China [3]. In Shandong, the HFRS epidemic exhibited a rebound trend, potentially due to changes associated with climate change and variations in rodent populations [4].

HFRS epidemics can be affected by environmental, population and reservoir factors, among which meteorological factors play an important role in the transmission of HFRS [5–10]. These meteorological factors, including temperature, precipitation and relative humidity, not only affect the transmission of Hantavirus but also impact the reservoir, rodents and contact chances between humans and rodents[11–13]. The infectivity and survival time of the Hantavirus after it leaves the host is largely dependent on the environmental temperature and humidity, and the chance of contact between humans and Hantavirus is influenced by the rainfall, temperature and humidity[11–13]. Few studies have investigated the impact of meteorological factors on the dynamics of HFRS in the context of the increasing trend in recent years.

Modeling and forecasting the HFRS epidemic is essential to controlling and preventing HFRS. Autoregressive Integrated Moving Average (ARIMA) models have been successfully applied to predict the incidence of infectious diseases, including HFRS [14,15] and other diseases [16–20]. Since HFRS presents typical seasonal characteristics [2,21–23], a Seasonal Autoregressive Integrated Moving Average (SARIMA) model can effectively simulate the HFRS epidemic.

In this study, we investigated seasonal HFRS variations and developed SARIMA models of the number of HFRS cases using time series analysis. The goal of this study was to characterize whether the inclusion of the affecting factors is useful in predicting epidemics with higher precision. The predictive model would be used to facilitate efficient HFRS control.

## Materials and Methods

### Study area

**Fig 1:**
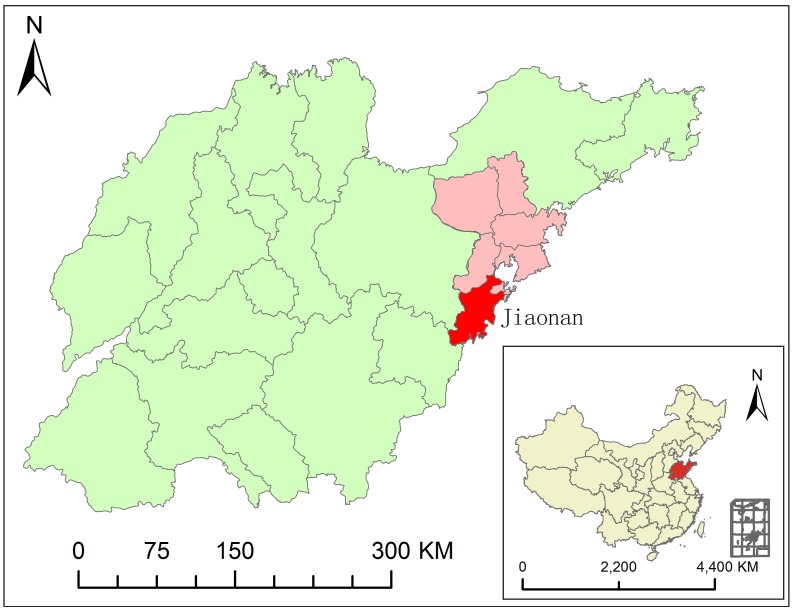
Geographical location of study area.

Jiaonan County is located in Qingdao, Shandong province (35°35′–36°08′N and 119°30′–120°11′E; Fig 1). The county is characterized by a coastal climate, with an average temperature of 12.1°C, annual precipitation of 750–900 mm and relative humidity of 75%.

### Data collection

Monthly HFRS epidemiologic data from Jiaonan were provided by the Jiaonan Center for Disease Control and Prevention, spanning from January 1992 to December 2014. Monthly meteorological data were interpolation data based on data retrieved from the China National Weather Data Sharing System (http://cdc.cma.gov.cn/home.do), which is publicly accessible. Meteorological data consisted of monthly mean temperature (Temp), monthly mean relative humidity (RH), and monthly mean precipitation (Prec) from 1992–2014.

### Statistical analyses

The analyses included descriptive and correlative approaches. The descriptive analysis included the number of cases, the long-term trends or seasonal variations and a summary of meteorological variables over the study period. A Spearman correlation analysis was performed between each meteorological variable and the number of cases. Moreover, given the potential lag effect of the meteorological variables on disease transmission, a cross-correlation analysis was also performed with relevant time lag values.

### Temporal simulation and validation

A SARIMA model was used to predict the number of HFRS epidemics in Jiaonan in this study. The SARIMA model was based on (p, d, q) (P, D, Q)[s], where p, d, and q are non-negative integers that indicate orders of non-seasonal AR terms, non-seasonal differencing and non-seasonal MA, respectively; P, D, Q are also non-negative integers that indicate orders of seasonal SAR terms, seasonal differencing and seasonal SMA terms, respectively; and s indicates the seasonal period (s = 12 months in this study).

The following steps were undertaken when modeling the number of HFRS epidemics and the meteorological variables. First, we used a mean range plot to determine whether the time series of HFRS and the climate variables exhibited stationary or non-stationary conditions. The autocorrelation function (ACF) and partial autocorrelation function (PACF) plots of HFRS in Jiaonan showed that it was non-stationary. Because both HFRS and the climate variables exhibited strong seasonal variations and fluctuations in their monthly means, we adjusted for seasonality by first seasonally differencing the series in the analysis. Second, the temporal structures of seasonal and non-seasonal autoregressive parameters (AR) (P, p) and moving average parameters (MV) (Q, q) in the series were determined by analysis of ACF and PACF analyses. Upon assessing the ACF and PACF results, the correlograms of the time series suggest that p and q should be <2 and d = 0. Third, parameters in the model were estimated using the maximum likelihood method, and the goodness-of-fit of each model was determined for appropriate modeling using the Ljung-Box test, measuring the ACFs and PACFs of the residuals, and checking the normality of the residuals. The significance of the parameters should be significantly different from zero. The normalized Bayesian Information Criterion (BIC) and stationary R square (R^2^) were also used to compare the goodness-of-fit among SARIMA models. The model with the lowest BIC and the highest stationary R^2^ values was considered a good model. The root mean square error (RMSE) was used to evaluate the predictive validity of the models. In this study, the data from January 1992 to December 2013 were used to construct a SARIMA model, and data from 2014 were used to validate the model.

We further evaluated whether alternative SARIMA models incorporating meteorological variables have greater predictive power. To overcome the autocorrelation within each individual series, the correlation coefficients between the number of HFRS epidemics and meteorological variables were computed after pre-whitening. Pre-whitening was performed by modeling each time series individually, using the SARIMA model to remove the trend and seasonal components and compute the correlation coefficients of the residuals of the time series. Climatic variables significantly associated with the number of HFRS cases were tested as predictors in the multivariate SARIMA model. A comparison of SARIMA with and without climatic variables was conducted. All statistical tests were 2-tailed, and a P value of 0.05 was considered statistically significant in terms of an explorative data analysis. For statistical analyses, we used SPSS software version 19 (SPSS).

## Results

### Statistical analyses

**Fig 2:**
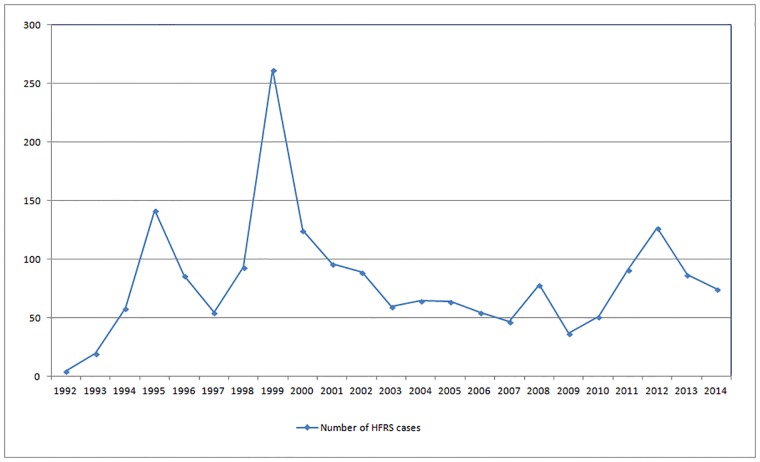
The number of HFRS cases in Jiaonan from 1992 to 2014.

**Fig 3:**
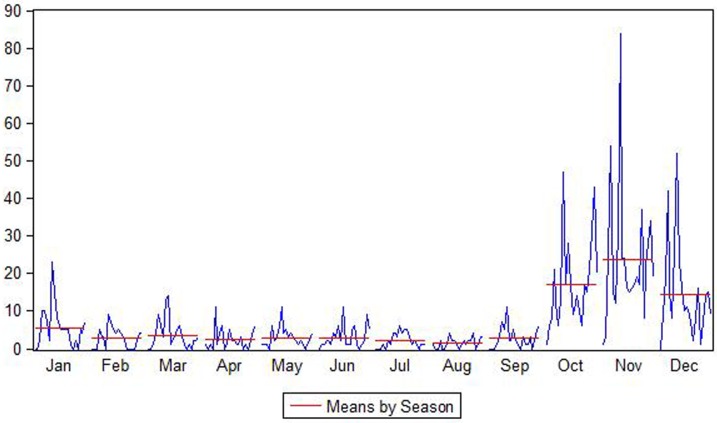
HFRS cases classified by season.

There were 1868 HFRS cases in Jiaonan from 1992–2014, and the number of cases fluctuated over the study period (Fig 2). The HFRS cases first increased and then decreased, with small fluctuations. The years with the most HFRS cases were 1995 (142 cases), 1999 (262 cases), 2008 (78 cases), and 2012 (127 cases). As shown in Fig 3, the months with the highest HFRS epidemic risk were November>October>December>January, with mean HFRS cases of 23.78>16.87>14.17>5.65.

**Fig 4:**
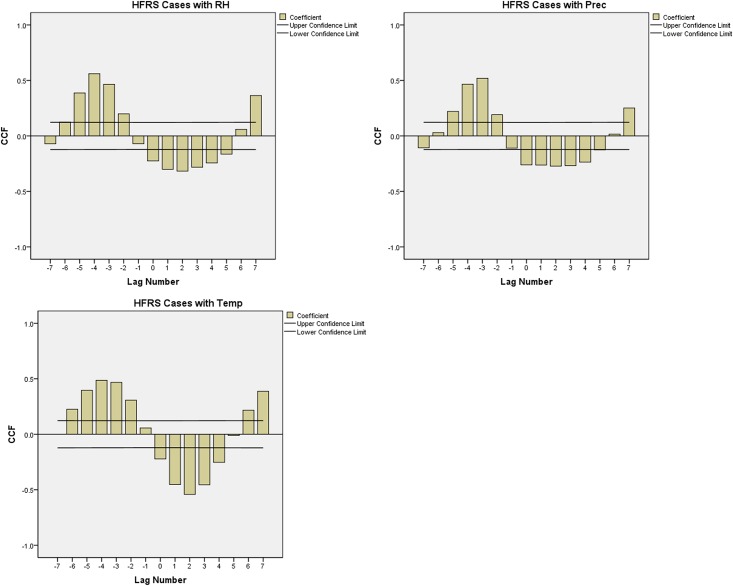
Cross-correlation analysis between HFRS cases and meteorological variables.

According to the Spearman correlation coefficients shown in Table 1, the number of HFRS cases was significantly associated with RH (-0.252**), Prec (-0.323**), Temp (-0.279**). According to the cross-correlation analysis (Fig 4), HFRS cases were negatively associated with 2 month-lagged RH, Prec, Temp, with coefficients of -0.317, -0.272, -0.542, respectively.

**Table 1:** Spearman Correlation coefficients of the association between HFRS cases and meteorological variables.

	HFRS Cases	RH	Prec	Temp
HFRS Cases	1	-.252**	-.323**	-.279**
RH	-.252**	1	.771**	.741**
Prec	-.323**	.771**	1	.749**
Temp	-.279**	.741**	.749**	1

** Correlation is significant at the 0.01 level (2-tailed).

### Temporal simulation using SARIMA models

#### Univariate SARIMA model

**Fig 5:**
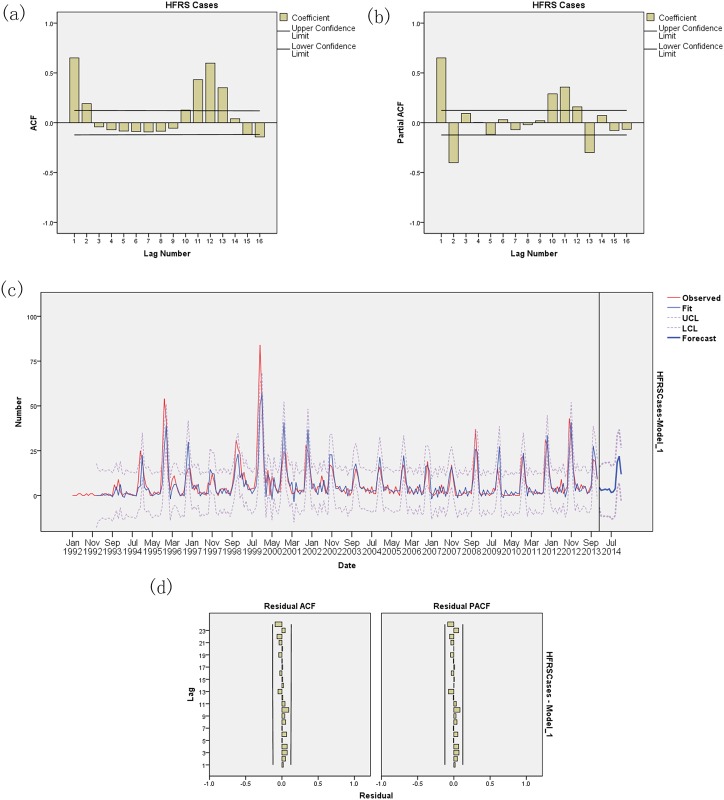
Univariate SARIMA analyses of HFRS cases. (a) Autocorrelation (ACF) plot of HFRS cases; (b) Partial ACF plot of HFRS cases; (c) SARIMA model of HFRS cases; (d) ACF and PACF plots of residuals after applying a SARIMA model.

The data were fitted with several univariate SARIMA models, and the models in which the residuals were not likely to be white noise were excluded. Among the models, the univariate SARIMA (0,0,2)(1,1,1)_12_ model had both the lowest BIC (3.628) and highest R^2^ (0.599) values (Fig 5, Table 2). Analyses of residuals in ACF and PACF plots assessed the absence of persistent temporal correlations. The Ljung-Box test confirmed that the residuals of time series were statistically independent. The selected model adequately fit observed data from 1992 to 2013. Furthermore, the model was used to forecast the number of HFRS cases between January and December 2014 and was validated using actual observations. The prediction of HFRS cases from January 2014 to December 2014 yielded an R^2^ value of 0.857 and RMSE of 2.708.

**Table 2:** Comparisons of univariate SARIMA models.

Model	R^2^	Ljung-Box Q statistics	Sig.	BIC	RMSE
SARIMA(0,0,2)(1,1,1)_12_	0.599	9.631	0.789	3.628	5.873
SARIMA(0,0,2)(0,1,1)_12_	0.585	20.340	0.159	3.663	5.974
SARIMA(1,0,2)(0,1,1)_12_	0.587	16.397	0.290	3.684	5.972
SARIMA(1,0,2)(1,1,1)_12_	0.600	9.224	0.756	3.678	5.890

#### Multivariate SARIMA model integrating meteorological variables

Next, we determined whether HFRS-associated meteorological variables could help refine the prediction model. To include meteorological variables (time series) as external variables, a multivariate SARIMA model was applied to the time series. To find the most HFRS-associated meteorological variables, a cross-correlation analysis was used to compute the lags of meteorological variables significantly associated with HFRS cases.

The BIC and RMSE values increased and R^2^ did not improve when the meteorological variables were considered external variables in the alternative SARIMA model (Table 3). Thus, the univariate SARIMA model predicted HFRS epidemics better than did the alternative SARIMA model that integrated meteorological variables.

**Table 3:** Multivariate SARIMA model integrating meteorological variables.

Model	R^2^	Sig.	BIC	RMSE
SARIMA(0,0,2)(1,1,1)_12_	0.6	0.722	3.617	5.785
with Prec	0.6	0.736	3.642	5.796
with RH	0.6	0.734	3.642	5.795
with Temp	0.6	0.716	3.641	5.792

## Discussion

In this paper, monthly HFRS cases in Jiaonan, Shandong, China, from 1992 to 2014 were modeled and validated using a SARIMA (0,0,2)(1,1,1)_12_ model. Furthermore, we tested whether meteorological variables, including temperature, precipitation and relative humidity, could be used to improve the prediction.

In mainland China, Shandong is one of the areas most severely affected by HFRS epidemics. Additionally, Qingdao City ranked first among cities with the most HFRS cases reported, and Jiaonan reported the most HFRS cases in Qingdao [24]. Although the incidence of HFRS is stable and exhibits a general decreasing trend at the national level in China, an increasing trend has been observed in Jiaonan in recent years [24].

HFRS is a disease with typical seasonal characteristics. In Jiaonan, most HFRS cases were observed in winter months (October, November, and December) and a spring month (January), and more cases were observed in the former season than in the latter season. According to a study of HFRS in China from 2006 to 2012 by Zhang et al., 65% of HFRS cases were reported in the spring and autumn-winter seasons [4], supporting the results of this study. Due to the seasonal variations in the HFRS epidemic, the SARIMA model can adequately simulate the HFRS epidemic.

In this study, meteorological variables, including relative humidity, precipitation and temperature, were correlated with HFRS cases to different degrees. According to a study in Jiaonan by Lin et al., meteorological variables, including daily temperature, humidity and rainfall, might be important predictors of HFRS epidemics in Jiaonan County [25], which has been verified in many studies.[11–13]. In this study, we tested whether meteorological variables (relative humidity, precipitation and temperature) significantly associated with HFRS cases could improve the SARIMA model as external regressors. The results showed that the meteorological variables did not significantly improve the SARIMA model, which can be explained as follows: (1) the seasonal variations in the meteorological variables were included in the seasonal mode characteristics of the HFRS itself and were hidden over a relatively large time span; and (2) Other factors, including vaccines, rodent control and improvement of living and working conditions, may affect HFRS epidemics.

Some limitations of this study should be noted. First, human rural activities, landscape features, land use, etc. can also affect HFRS occurrence; however, due to data availability and regain cycles, they were not included in this study. Second, vaccine inoculation has an important impact on HFRS epidemics and may directly affect HFRS epidemics in the subsequent months and years; thus, inoculation should be included in the SARIMA model to improve the prediction precision in the future.

The SARIMA model is good at simulating the temporal variations in HFRS epidemics in China, which should aid hygienic authorities in creating effective measures that prevent and control this disease. More temporal models should incorporate powerful environmental factors to synthetically predict HFRS epidemics in the future.

## Conclusion

The SARIMA model developed in this study can be used as an early and reliable monitoring system to predict annual HFRS epidemics. Climate patterns and HFRS were highly correlated; however, they did not improve the simulation results when included in the SARIMA model. The result was likely because the seasonal variations in meteorological variables were included in the seasonal mode characteristics of the HFRS itself.
